# LncRNA NR2F2‐AS1 promotes tumourigenesis through modulating BMI1 expression by targeting miR‐320b in non‐small cell lung cancer

**DOI:** 10.1111/jcmm.14102

**Published:** 2018-12-27

**Authors:** Shijie Zhang, Xiaoyun Zhang, Qianqian Sun, Chunbo Zhuang, Guanlin Li, Li Sun, Huaqi Wang

**Affiliations:** ^1^ Department of Clinical Laboratory The First Affiliated Hospital of Zhengzhou University Zhengzhou China; ^2^ Department of Clinical Laboratory Zhengzhou Children’s Hospital, Henan Children’s Hospital, Children’s Hospital Affiliated of Zhengzhou University Zhengzhou China; ^3^ Department of Respiratory Medicine The First Affiliated Hospital of Zhengzhou University Zhengzhou China

**Keywords:** apoptosis, BMI1, invasion, LncRNA NR2F2‐AS1, miR‐320b, NSCLC, proliferation

## Abstract

Recently, long noncoding RNAs (lncRNAs) are attracting wide attention in the field of cancer research because of its important role in cancer diagnosis and prognosis. But studies on the biological effects and relevant mechanisms of lncRNAs in non‐small cell lung cancer (NSCLC) remain few and need to be enriched. Our study discussed the expression and biological effects of LncRNA NR2F2‐AS1, and further explored its possible molecular mechanisms. As a result, elevated expression of NR2F2‐AS1 was detected in NSCLC tissues and cells and was remarkably associated with the tumor, node, metastasis (TNM) stage and the status of lymphatic metastasis of patients. Down‐regulated NR2F2‐AS1 contributed to the promotion of cell apoptosis and the inhibition of cell proliferation and invasion in A549 and SPC‐A‐1 cells in vivo and vitro. Through bioinformatics analysis, NR2F2‐AS1 functions as a ceRNA directly binding to miR‐320b, BMI1 was a direct target of miR‐320b. Combined with the following cellular experiments, the data showed that NR2F2‐AS1 may influence the NSCLC cell proliferation, invasion and apoptosis through regulating miR‐320b targeting BMI1.

## 1 INTRODUCTION

Lung cancer is the most commonly reported cancers worldwide, the new cases diagnosed are increasing year by year.^1^ Despite the fact that comprehensive treatment, including surgical resection, radiotherapy and chemotherapy, continues to develop, the overall 5‐year survival rate of lung cancer patients is less than 20%^2^ which has not been obviously improved over the past few decades. Non‐small cell lung cancer (NSCLC) is a main histological type of lung cancer with a constituent per cent of approximately 85%.^3^ Notably, the proportion of patients who undergo surgical resection for NSCLC is in low level, for most of the patients are usually diagnosed at an advanced stage. As a result, improving the diagnosis and treatment of NSCLC really needs more attention. In the process of cancer development, multiple factors are involved and may influence treatment and prognosis. It is necessary to further understand relevant molecular mechanisms of NSCLC so that we can discover potential therapeutic targets which are helpful for cancer diagnosis or limiting cancer progress.

Recently, long noncoding RNAs (lncRNAs) are attracting wide attention in the field of cancer research because of its important role in cancer diagnosis and prognosis.^4^ LncRNAs itself are defined as non‐protein coding transcripts longer than 200 nucleotides.^5^, ^6^, ^7^ Research shows that abnormal expression of lncRNAs occurs in various cancer diseases,^8^ including liver cancer,^9^ gastric cancer,^10^ oesophageal cancer,^11^ breast cancer and prostate cancer.^12^ Also, this kind of phenomenon can be found in coronary artery disease,^13^ and nerve system disease.^14^ Through the latest technology including bioinformatics analyses and next‐generation sequencing technology, the regulatory effects and potential application of lncRNAs were slowly and partly come into our visual fields, such as lncRNA LINC01116, CASC9, ANRIL.^15^, ^16^, ^17^, ^18^ In the NSCLC which we have paid attention to, articles have demonstrated that long non‐coding RNA MEG3 could inhibit NSCLC cell proliferation and induces apoptosis by affecting p53 expression^19^; lncRNA AK126698 can affect the cisplatin resistance of NSCLC cells.^20^ As a result, it is well worth exploring the LncRNAs and their relative mechanisms in NSCLC.

In this study, LncRNA NR2F2‐AS1 was found to be abnormally expressed based on the previous analysis of lncRNAs microarray among NSCLC tissues.^21^ NR2F2, also named COUP‐TFII, belongs to the nuclear receptor family and has been reported to be important in the treatment and prevention of cancers.^22^, ^23^ So far, the function of LncRNA NR2F2‐AS1 in NSCLC has not been explored. We aimed to confirm the expression of NR2F2‐AS1 in NSCLC and dig into relevant mechanisms of its regulatory network.

## 2 MATERIALS AND METHODS

### 2.1 Patients and tissue specimens

For this study, we spent about a year to collect relative tissues. Thirty‐nine paired of tissue specimens and matched normal tissue samples obtained from the NSCLC patients underwent operation at the First Affiliated Hospital of Zhengzhou University from March 2016 to August 2017. The tissues were stored in liquid nitrogen until the isolation of total RNA. In this cohort of NSCLC patients, 22 were men and 17 were women, with a median age of 56 years. According to the 7th TNM staging system,^24^ 23 cases of the NSCLC patients were classified as stage I/II and 16 cases were classified as stage III. Among all of the cases, 28 had lymph node metastasis. Before we carried out this study, all the patients were informed and the informed consent forms were duly signed. Moreover, The Ethics Review Committee of Zhengzhou University approved the study.

### 2.2 Cell culture and transfection

We purchased lung cancer cell lines including A549, H460, H1299, SPC‐A‐1, Calu‐3 and H1650 cells from ATCC (Manassas, VA, USA). The normal human bronchial epithelial (NHBE) cell line was preserved in our laboratory. All the cells were cultured in the DMEM medium (Gibco, MA, USA) with 10% FBS (Gibco) and 1% penicillin‐streptomycin (Gibco) in an atmosphere of 5% CO_2 _at 37°C.

In our study, si‐LncRNA NR2F2‐AS1, miR‐320b mimic and si‐BMI1 (GenePharma, Shanghai, China) were transfected, or cotransfected into cells according to the experimental aims. To be specific, cells were cultured in the 6‐well plates. When the confluence was approximately 60%–70%, the medium was changed to DMEM without 10% FBS and the transfection was performed using Lipofectamine™ 3000 (Invitrogen, CA, USA). After 24–72 hours of transfection, cells were harvested to the following operation. The sequence of si‐LncRNA NR2F2‐AS1 was 5ʹ AAGGATGTCAGCGCACTAAAT 3ʹ; the sequence of miR‐320b mimic was 5ʹ AAAAGCUGGGUUGAGAGGGCAA 3ʹ and 5ʹ GCCCUCUCAACCCAGCUUUUAU 3ʹ; the sequence of si‐BMI1 was 5ʹ CCAAGAUAUUGUAUACAAAUU 3ʹ and 5ʹ UUUGUAUACAAUAUCUUGGAG 3ʹ.

### 2.3 RNA isolation and quantitative real time PCR

Total RNA in NSCLC and normal tissues was extracted using TRIzol reagent (Invitrogen, USA). The uptake of total RNA in lung cancer cells was performed through an EZNA Total RNA Kit I (Omega Bio‐Tek, Inc, GA, USA) The concentration and quality of RNA was measured by Nanodrop 2000 spectrophotometry (Thermo Fisher Scientific, CA, USA). After being reverse‐transcribed to cDNA with the use of a PrimeScript RT reagent Kit (Takara, China), qRT‐PCR (quantitative real‐time PCR) was carried out to detect the expression of LncRNA NR2F2‐AS1 and miR‐320b with the assistance of 7500 Fast PCR instrument (Applied Biosystems, MA, USA). For normalization, GAPDH or U6 was regarded as an endogenous reference. Fold change of gene expression was expressed by the 2^–△△Ct^ method. In the qRT‐PCR experiment, the following were used for LncRNA NR2F2‐AS1 primer: Forward primer: 5ʹ TCAGCCGGAAAACTACAAGCTC 3ʹ and Reverse primer: 5ʹ TCTTCGTGTAGCTGTTCCACC 3ʹ; miR‐320b primer: RT primer: 5ʹ GTCGTATCCAGTGCAGGGTCCGAGGTATTCGCACTGGATACGACTTTTCGAC 3ʹ, Forward primer: 5ʹ TCCGAAACGGGAGAGTTGG 3ʹ and Reverse primer: 5ʹ GTGCAGGGTCCGAGGT 3ʹ; GAPDH primer: Forward primer: 5ʹ GCACCGTCAAGGCTGAGAAC 3ʹ and Reverse primer: 5ʹ TGGTGAAGACGCCAGTGGA 3ʹ. U6 primer: RT primer: 5ʹ GTCGTATCCAGTGCAGGGTCCGAGGTATTCGCACTGGATACGACAAAATA 3ʹ Forward primer: 5ʹ TCCGATCGTGAAGCGTTC 3ʹ, Reverse primer: 5ʹ GTGCAGGGTCCGAGGT 3ʹ.

### 2.4 Cell apoptosis detection

FCM (Flow cytometry) is one way to analyse the condition of cell apoptosis. For this, we purchased an Annexin V‐FITC/PI Apoptosis Detection Kit from Solarbio (Beijing, China). Firstly, cells were treated and cultured in a 6‐well plate for 24 hours. Next, cells were washed with 1 × PBS (Solarbio, Beijing, China), and resuspended in binding buffer (500 μL) in a 1.5 mL Eppendorf tube. Finally, cells were double‐stained with Annexin V‐FITC and PI (5 μL) for 5–15 minutes at room temperature and identified using a FACScan^®^ flow cytometer equipped with CellQuest software (BD Biosciences, San Jose, CA). What calls for special attention is that cells should be kept away from light when being stained.

Caspase3/7 activity detection was another accessible way to measure cell apoptosis using the Caspase‐Glo^®^ 3/7 assay (Promega Corporation, Madison, WI, USA). In A549 and SPC‐A‐1 cells, miR‐320b mimic group (cells transfected with miR‐320b mimic) and miR‐NC group (cells transfected with miR‐320b scramble) were seeded in 96‐well plates then, the cell culture medium accompanied by Caspase‐Glo^®^ 3/7 reagent was added to the plates and cells were incubated for 4 hours at 37˚C. The luminescence (an excitation wavelength of 490 nm and an emission wavelength of 530 nm) was then detected in a luminometer plate reader.

### 2.5 Cell proliferation assessments in vitro and vivo

CCK‐8 (Cell counting Kit‐8) assay is an usual method to evaluate cell viability and was applied in our study to explore the factors influencing cell proliferation in A549 and SPC‐A‐1 cells. The two cells were seeded in a 96‐well plate at a density of 1×10^4^ cells respectively. After treatment and incubation for 0, 24, 48 and 72 hours, 10 µL of CCK‐8 kit (Djingo, Japan) was added to each well. Finally, the optical density (OD) value was quantified using an automatic microplate reader (Synergy4, BioTek, Winooski, VT) at a wavelength of 450 nm after incubation at 37°C for 2 hours. The experiments were repeated in triplicate independently to explore cell proliferation in vitro.

In order to further investigate whether down‐regulation of LncRNA NR2F2‐AS1 influences the growth of tumour cells, 20 BALB/c nude mice (4‐week‐old, female) were purchased from Beijing Vital River Laboratory Animal Technology Center (Beijing, China) and were divided into four groups according to cell transfection. The A549 and SPC‐A‐1 cells in which firefly luciferase was stably expressed were purchased from PerkinElmer (Waltham, MA, USA). si‐LncRNA NR2F2‐AS1 or si‐NC transfected cells were subcutaneously implanted into the left armpit area at a concentration of 2×10^6^ cells. An in vivo small animal imaging instrument (PerkinElmer, Waltham, MA, USA) was used weekly to monitor the luciferase signal of the mice. In the fourth week, all these mice were dissected after cervical dislocation. The tumour weight was then calculated with the electronic balance. The whole study was approved by the Animal Care and Use Committee of the Zhengzhou University.

### 2.6 Cell invasion assays

For the sake of cell invasion exploration, a transwell assay was performed in A549 and SPC‐A‐1 cells. Cells were transfected on the basis of experimental aims. Then, 5×10^5^ cells/mL cells were incubated in the upper chamber of a 24‐well Transwell Permeable Support with 100 μL serum‐free DMEM, and the bottom chambers were filled with medium containing 10% FBS. Cells in the upper chamber slowly moved to the lower chamber through Matrigel over time. After 48 hours, the membrane containing invaded cells was soaked in methanol and stained with 0.1% crystal violet. Cells were finally photographed and calculated under an inverted microscope (Olympus, Japan). The experiments were repeated in triplicate independently.

### 2.7 Dual‐luciferase assays

In order to investigate the relationship between LncRNA NR2F2‐AS1 and miR‐320b as well as miR‐320b and BMI1, we first searched the potential binding sites through the informatics analysis of microRNA database and Target Scan. PCR and overlap PCR were performed to amplify LncRNA NR2F2‐AS1 cDNA containing predicted miR‐320b binding site or the 3ʹUTR of BMI1 and then the PCR products were cloned downstream of the luciferase gene in the pmirGLO vector. When it comes to the dual‐luciferase assay, the combined plasmids pmirGLO‐Wt‐LncRNA NR2F2‐AS1 or pmirGLO‐Mt‐LncRNA NR2F2‐AS1 were transiently cotransfected to A549 and SPC‐A‐1 cells accompanied by miR‐320b mimic or scrambled miRNA (miR‐NC) through Lipofectamine™ 3000 (Invitrogen, CA, USA), which is similar to the cotransfection of BMI1 combined plasmids and miR‐320b mimic or miR‐NC. Finally, the relative luciferase activity was determined with the luciferase assay kit (Promega, USA) at 48 hours after transfection.

### 2.8 Western blot assays

We use Western blot assays to measure the expression of BMI1 protein level in A549 and SPC‐A‐1 cells. The RIPA lysis buffer (Solarbio, Beijing, China) was used to lyse cells to obtain total protein which was quantified by a BCA protein assay kit (Beyotime, Shanghai, China). About 50 µg of protein was separated by electrophoresis with 10% SDS‐PAGE and transferred onto a PVDF membrane (Thermo Scientific, MA, USA). After being blocked in 5% skim milk powder for 1 hour, the membrane was probed with the primary antibody for BMI1 and GAPDH at a concentration of 1:500 and 1:1000, respectively at 4°C overnight. The next day the membrane was washed with TBST for four times (5, 10, 10, 15 minutes) at room temperature. The membrane was then incubated with the secondary antibody for 1 hour at a concentration of 1:5000. Wash it again with TBST for four times. Finally, the ECL Substrate (Thermo Scientific, MA, USA) was added to determine the protein immunoreactivity by FluorChem E (ProteinSimple, CA, USA).

### 2.9 Statistical analysis

The whole data obtained above were analysed using the SPSS software version 22.0. Mean ± Standard deviation (SD) is the way to present the results of the statistical analysis. According to the data types, Student's *t* test and one‐way ANOVA were carried out to compare statistical significance among groups. When it comes to evaluate the correlation between two variants, the spearman rank correlation test was applied. A *P *< 0.05 was considered statistically significance.

## 3 RESULTS

### 3.1 The expression of LncRNA NR2F2‐AS1 was in high level in NSCLC tissues and cells

**Figure 1:**
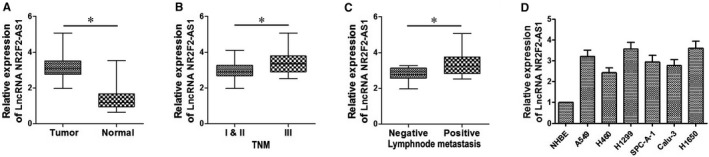
LncRNA NR2F2‐AS1 expression in non‐small cell lung cancer (NSCLC) tissues and cells. A, qRT‐PCR analysis of NR2F2‐AS1 levels in 39 paired NSCLC and normal tissue samples. (B, C) the statistical analysis of the relationship between LncRNA NR2F2‐AS1 expression and clinicopathological features (TNM stage and lymphatic metastasis) in patients suffering from NSCLC. D, qRT‐PCR analysis of NR2F2‐AS1 levels in normal human bronchial epithelial (NHBE) cells and A549, H460, H1299, SPC‐A‐1, Calu‐3 and H1650 cancer cells. *P < 0.05

In order to disclose the role of LncRNA NR2F2‐AS1 in NSCLC carcinogenesis, the expression of LncRNA NR2F2‐AS1 in NSCLC samples and cell lines was analysed through qRT‐PCR. As shown in Figure 1A and Table 1, LncRNA NR2F2‐AS1 expression in 39 surgically resected NSCLC tissues was significantly higher than that of the corresponding normal tissues (*P < *0.05). Based on this finding, we carried out the statistical analysis in the field of age, gender, tumour stage (according to the 7th TNM staging system^24^) and the condition of lymphatic metastasis to futher investigate the relationship between LncRNA NR2F2‐AS1 expression and clinicopathological features in patients suffering from NSCLC. The results obviously demonstrated that the level of LncRNA NR2F2‐AS1 was up‐regulated in patients with advanced TNM stage (*P < *0.05, Figure 1B) or positive lymphatic metastasis (*P < *0.05, Figure 1C). Additionally, we detected the expression of LncRNA NR2F2‐AS1 in six kinds of NSCLC cells, and the normal human bronchial epithelial cells (NHBE) served as control. We found that LncRNA NR2F2‐AS1 levels in A549, H460, H1299, SPC‐A‐1, Calu‐3 and H1650 cells were all up‐regulated (*P < *0.05, Figure 1D) compared to that in NHBE cells. As a result, we summarized that the expression of LncRNA NR2F2‐AS1 was in high level in NSCLC tissues and cells and was remarkably associated with the TNM stage and the status of lymphatic metastasis of patients.

**Table 1:** Clinicopathological characteristics and LncRNA NR2F2‐AS1 expression levels in 39 non‐small cell lung cancer (NSCLC) patients

Clinicopathological parameters	Cases	LncRNA NR2F2‐AS1 expression	
		Mean ± SD	*P *value
Age (y)			
≤56	20	3.02 ± 0.47	0.177
＞56	19	3.27 ± 0.67	
Gender			
Male	22	3.21 ± 0.57	0.752
Female	17	3.14 ± 0.65	
TNM stage			
I + II	23	3.43 ± 0.64	0.029^a^
III	16	2.99 ± 0.51	
Lymph node metastasis			
Negative	11	2.74 ± 0.41	0.015^a^
Positive	28	3.35 ± 0.59	

a Indicates significant differences (*P* < 0.05).

### 3.2 Down‐regulated LncRNA NR2F2‐AS1 contributed to the promotion of cell apoptosis and the inhibition of cell proliferation and invasion in A549 and SPC‐A‐1 cells

**Figure 2:**
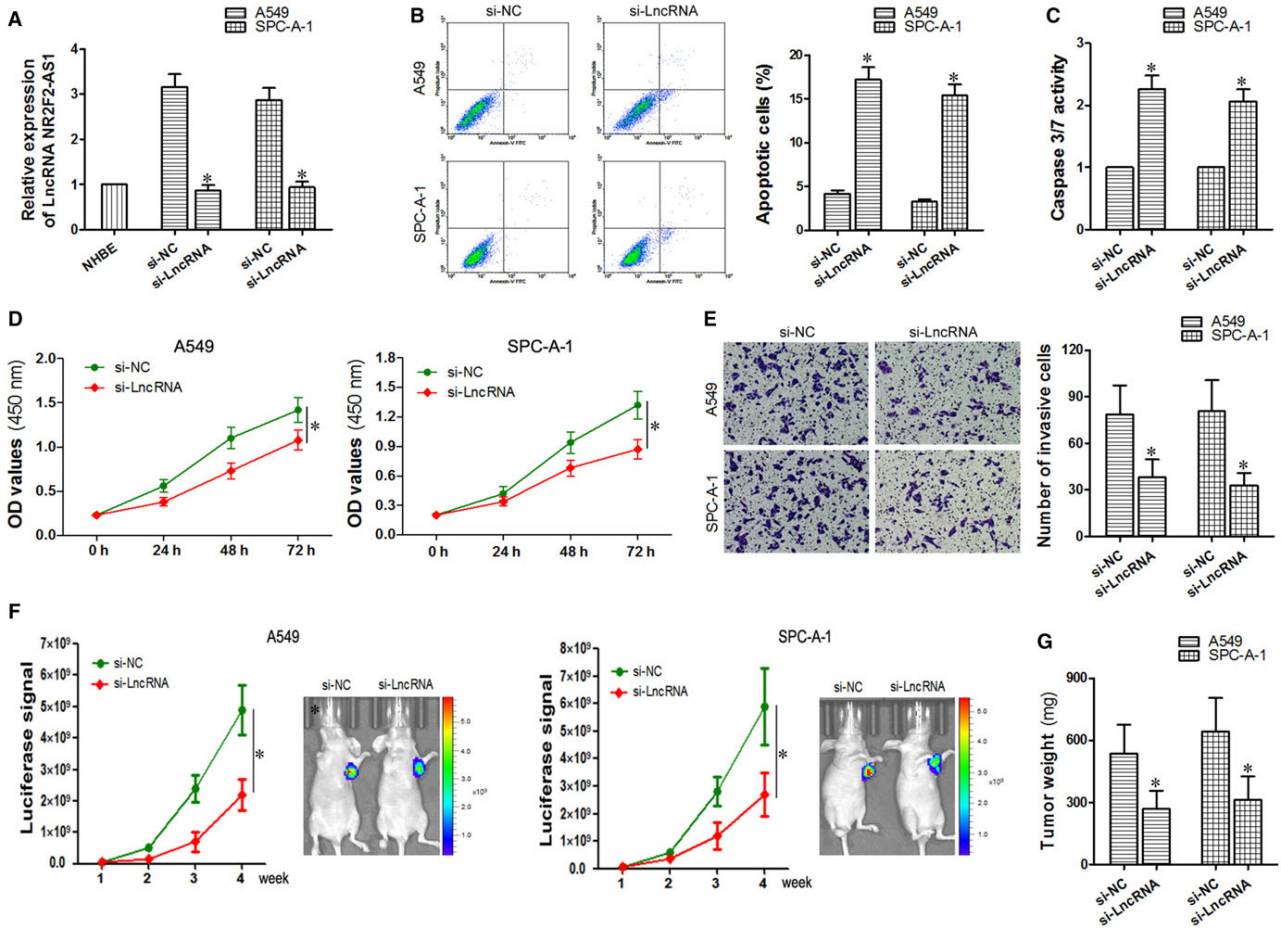
Effects of down‐regulated LncRNA NR2F2‐AS1 on cell apoptosis proliferation and invasion in A549 and SPC‐A‐1 cells. A, detection of si‐LncRNA NR2F2‐AS1 transfected effects through qRT‐PCR. (B, C), flow cytometry and caspase 3/7 activity assay were used to evaluate the effects of down‐regulated NR2F2‐AS1 on cell apoptosis. D, CCK‐8 assay was performed to analyse the effects of down‐regulated NR2F2‐AS1 on cell proliferation. E, transwell assay was carried out to study the effects of down‐regulated NR2F2‐AS1 on cell invasion. (F, G), xenograft experiments in nude mice were used to further search the effects of down‐regulated NR2F2‐AS1 on cell proliferation in vivo. *P < 0.05

In the previous study, the expression of LncRNA NR2F2‐AS1 was confirmed to be up‐regulated in NSCLC tissues and cells. Next, we wonder to explore if the down‐regulation of LncRNA NR2F2‐AS1 could interfere NSCLC progression. Si‐LncRNA NR2F2‐AS1 was transfected into A549 and SPC‐A‐1 cells, and the results of qRT‐PCR showed that the expression of LncRNA NR2F2‐AS1 was successfully inhibited through the transfection (*P < *0.05, Figure 2A). As a result, the A549 and SPC‐A‐1 cells were both divided into two groups, that is si‐ LncRNA group (LncRNA NR2F2‐AS1 was in low level) and si‐NC group (the negative control group). The results of the two groups in cell apoptosis assay, cell proliferation detection and cell invasion assay could be seen in Figure 2B‐E. Compared with the si‐NC group, apoptotic cells in si‐LncRNA group was significantly in high level (*P < *0.05); on the contrary, the A450 value in si‐LncRNA group was obviously decreased(*P < *0.05); similarly, the number of invasive cells was reduced in si‐LncRNA group (*P < *0.05). These experiments above proved that down‐regulated LncRNA NR2F2‐AS1 could promote cell apoptosis, while inhibiting cell proliferation and invasion in vitro. Moreover, models of transplanted tumour on nude mouse were used to study the effect in vivo. As shown in Figure 2F, the luciferase signal in si‐LncRNA group was typically lower than the si‐NC group (*P < *0.05), similarly, the tumor weight in si‐LncRNA group was obviously lower than the si‐NC group (*P < *0.05, Figure 2G).Taken together, we concluded that down‐regulated LncRNA NR2F2‐AS1 contributed to the promotion of cell apoptosis and the inhibition of cell proliferation and invasion in A549 and SPC‐A‐1 cells in vivo and vitro.

### 3.3 LncRNA NR2F2‐AS1 functions as a ceRNA directly binding to miR‐320b

**Figure 3:**
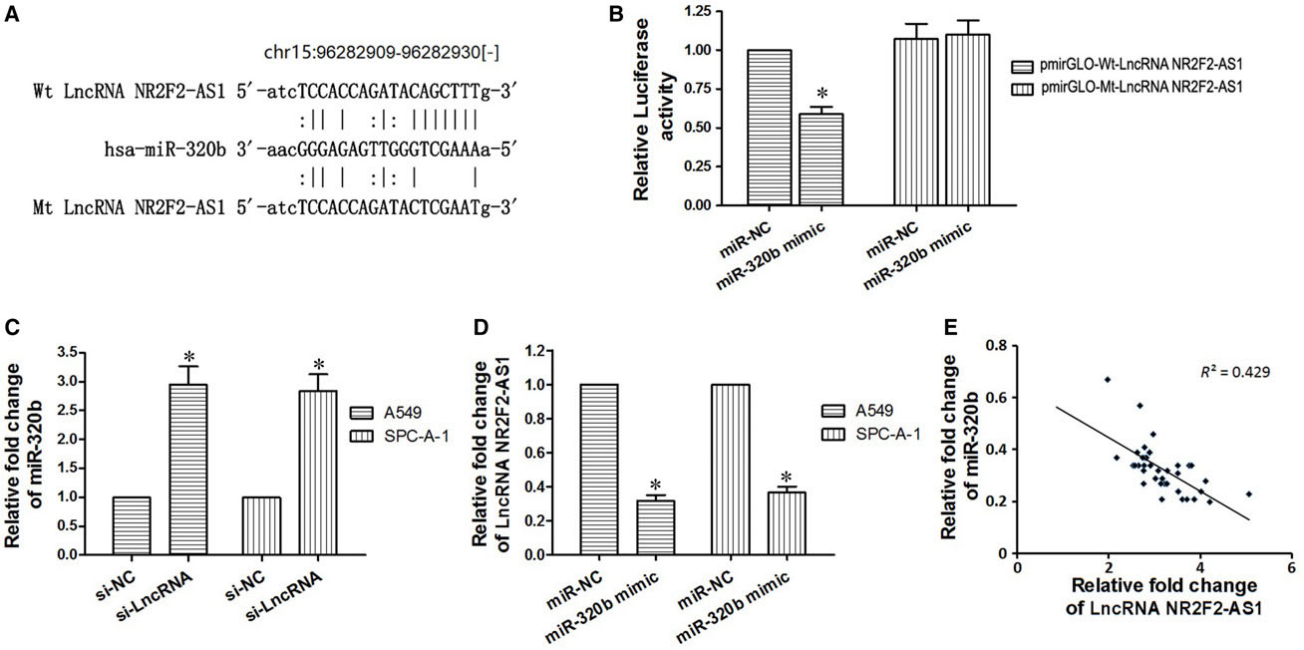
LncRNA NR2F2‐AS1 could act as a ceRNA through binding to miR‐320b. A, The bioinformatics analysis of microRNA database of NR2F2‐AS1. B, Relative luciferase activity is decreased in cells transfected with pmirGLO‐wt‐LncRNA NR2F2‐AS1 and miR‐320b mimic than in cells transfected with Mt‐NR2F2‐AS1 and miR‐320b mimic, demonstrating that miR‐320b directly binds to NR2F2‐AS1. C, down‐regulated NR2F2‐AS1 could lessen the expression of miR‐320b. D, up‐regulated miR‐320b could inhibit the expression of NR2F2‐AS1. E, Pearson's analysis shows correlation between NR2F2‐AS1 and miR‐320b levels in tissue samples (n = 39, Pearson's coefficient [*R*] = –0.655). *P < 0.05

Salmena et  al^25^ presented ceRNA (competing endogenous RNAs) hypothesis which stated that the pool of mRNAs, lncRNAs, and other non‐coding RNAs shared common MREs with miRNAs, which can serve as natural miRNA “sponges” and can inhibit the miRNA function through competitively binding to MREs on the target mRNA. In this study, we supposed there existed one or more miRNAs which could be regulated through the function of LncRNA NR2F2‐AS1. The bioinformatics analysis of microRNA database found the putative binding sites between LncRNA NR2F2‐AS1 and miR‐320b (Figure 3A). To further test this kind of relationship, a luciferase report assay was performed and as seen in Figure 3B, miR‐320b mimic lessened the luciferase activity of pmirGLO‐wt‐LncRNA NR2F2‐AS1 but not of pmirGLO‐wt‐LncRNA NR2F2‐AS1 (*P < *0.05). Moreover, we examined the expression level of miR‐320b in A549 and SPC‐A‐1 cells transfected with si‐LncRNA NR2F2‐AS1 and the level of LncRNA NR2F2‐AS1 in cells transfected with miR‐320b mimic respectively. The results showed that miR‐320b was significantly up‐regulated when decreasing the expression of LncRNA NR2F2‐AS1 (*P < *0.05, Figure 3C); in turn, when we artificially elevated the expression of miR‐320b, LncRNA NR2F2‐AS1 expression was in a relatively low level (*P < *0.05, Figure 3D). Based on the inverse relationship between LncRNA NR2F2‐AS1 and miR‐320b (*R* ^2^ = 0.429, *P < *0.05, Figure 3E), we concluded that in A549 and SPC‐A‐1 cells, LncRNA NR2F2‐AS1 could act as a ceRNA through binding to miR‐320b.

### 3.4 Up‐regulated miR‐320b inhibited cell proliferation and invasion and induce cell apoptosis in A549 and SPC‐A‐1 cells

**Figure 4:**
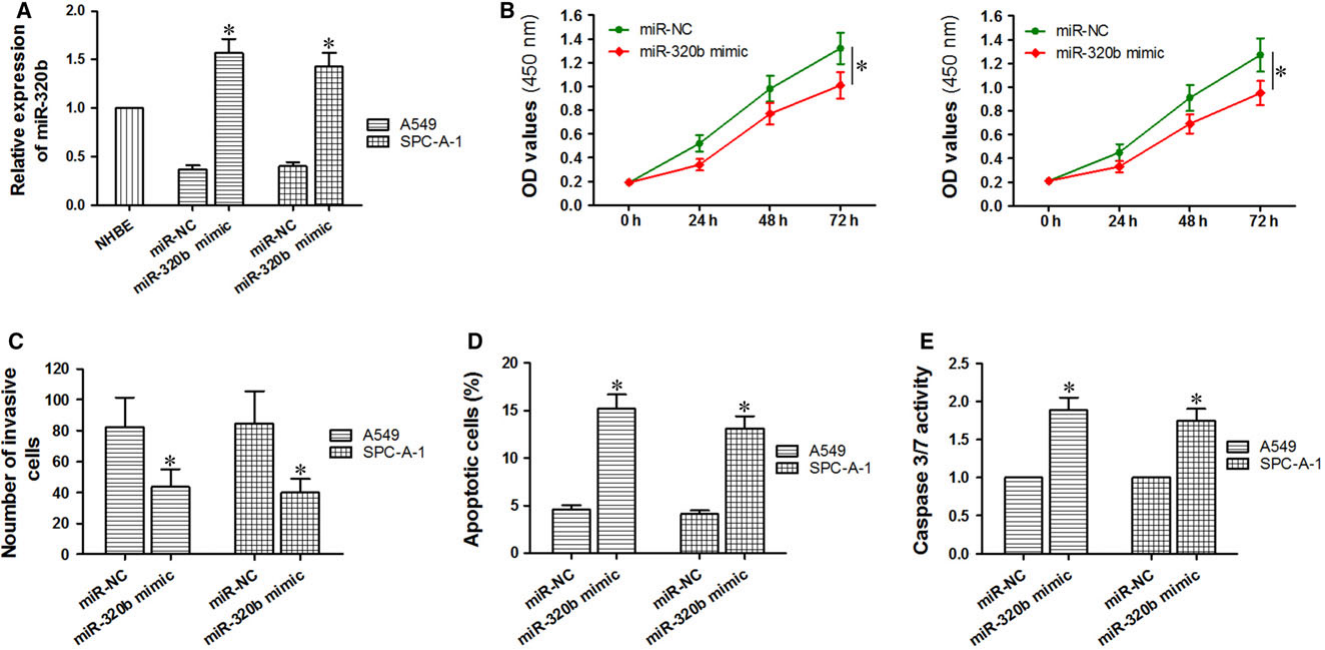
Effects of up‐regulated miR‐320b on cell apoptosis, proliferation and invasion in A549 and SPC‐A‐1 cells. A, detection of miR‐320b mimic transfected effects through qRT‐PCR. B, CCK‐8 assay was performed to analyse the effects of up‐regulated miR‐320b on cell proliferation. C, transwell assay was carried out to study the effects of up‐regulated miR‐320b on cell invasion. (D, E) flow cytometry and Caspase 3/7 activity assay were used to evaluate the effects of up‐regulated miR‐320b on cell apoptosis. *P < 0.05

Based on the data obtained above, we have found that when LncRNA NR2F2‐AS1 was down‐regulated in A549 and SPC‐A‐1 cells, miR‐320b was up‐regulated accordingly; Down‐regulated LncRNA NR2F2‐AS1 contributed to the promotion of cell apoptosis and the inhibition of cell proliferation and invasion. Then, we wonder how to explore the effects of overexpressed miR‐320b in lung cancer cells. As shown in the Figure 4A, the expression of miR‐320b was successfully increased after being transfected with miR‐320b mimic. Compared with the NC group, the A450 value of miR‐320b mimic group was obviously decreased (*P < *0.05, Figure 4B) along with the number of invasive cells (*P < *0.05, Figure 4C). On the contrary, the number of apoptotic cells in miR‐320b mimic group was significantly increased than that of the NC group (*P < *0.05, Figure 4D). To further confirm the condition of cell apoptosis, the caspase3/7 activity was also detected, and the results were consistent with the FCM assay (*P < *0.05, Figure 4E). Through the above experiments , up‐regulated miR‐320b turned out to inhibit cell proliferation and invasion and promote cell apoptosis in A549 and SPC‐A‐1 cells.

### 3.5 BMI1 was a direct target of miR‐320b

**Figure 5:**
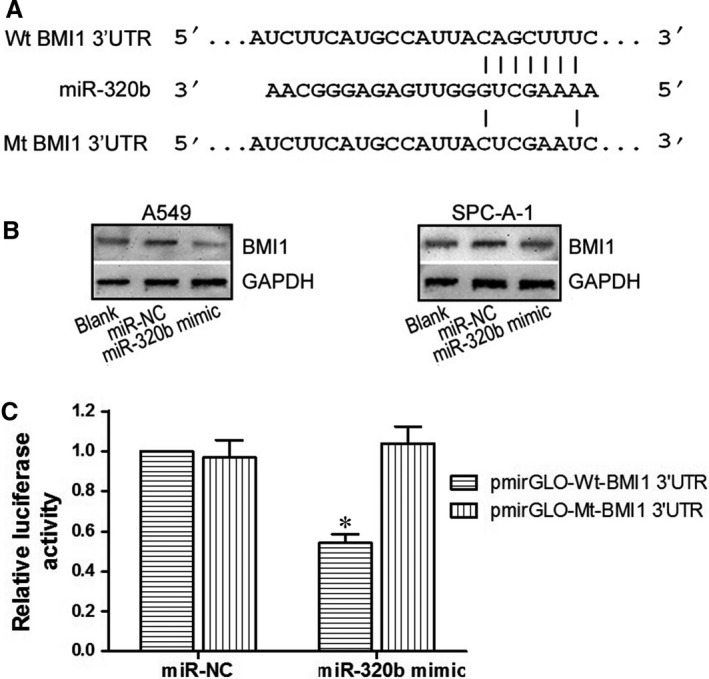
BMI1 was a direct target of miR‐320b. A, The bioinformatics analysis of microRNA database and Target Scan of miR‐320b. B, Western blot assay of BMI1 protein expression in miR‐320b mimic, miR‐NC and blank groups. C, Relative luciferase activity is decreased in cells transfected with pmirGLO‐Wt‐BMI1 3ʹUTR and miR‐320b mimic than in cells transfected with Mt‐BMI1 3ʹUTR and miR‐320b mimic, demonstrating that miR‐320b directly binds to BMI1. *P < 0.05

In order to elucidate the possible mechanisms of miR‐320b in the apoptosis, proliferation and invasion of A549 and SPC‐A‐1 cells, the bioinformatics analysis of microRNA database and Target Scan was applied to search the putative binding sites of target genes. As indicated in Figure 5A, miR‐320b may regulate BMI1 by binding to its 3ʹUTR. To identify this hypothesis, a mutant BMI1 3ʹUTR was designed (Figure 5A) and amplified using PCR. After successfully constructing wild and mutant type of BMI1 3ʹUTR vector (pmirGLO‐Wt‐BMI1 3ʹUTR and pmirGLO‐Mt‐BMI1 3ʹUTR), the dual‐luciferase report assay was performed in A549 and SPC‐A‐1 cells. As a consequence, a significant decrease in the relative luciferase activity was identified following cells co‐transfected with pmirGLO‐Wt‐BMI1 3’UTR plasmid and miR‐320b mimic. (*P*<0.05, Fig.5C); moreover, when cells were co‐transfected with pmirGLO‐Mt‐BMI1 3’UTR and miR‐320b mimic or co‐transfected with pmirGLO‐Mt‐BMI1 3’UTR and miR‐NC, the relative luciferase activity in the two groups showed no difference (*P > *0.05, Figure 5C). The expression of BMI1 protein was also measured using Western Blot assay and was suppressed by overexpression of miR‐320b (*P < *0.05, Figure 5B). All these results indicated that miR‐320b could down‐regulate the expression of BMI1 for the fact that BMI1 is a direct target of miR‐320b.

### 3.6 Down‐regulated LncRNA NR2F2‐AS1, up‐regulated miR‐320b and si‐BMI1 shared similar effects in cell proliferation, invasion and apoptosis

**Figure 6:**
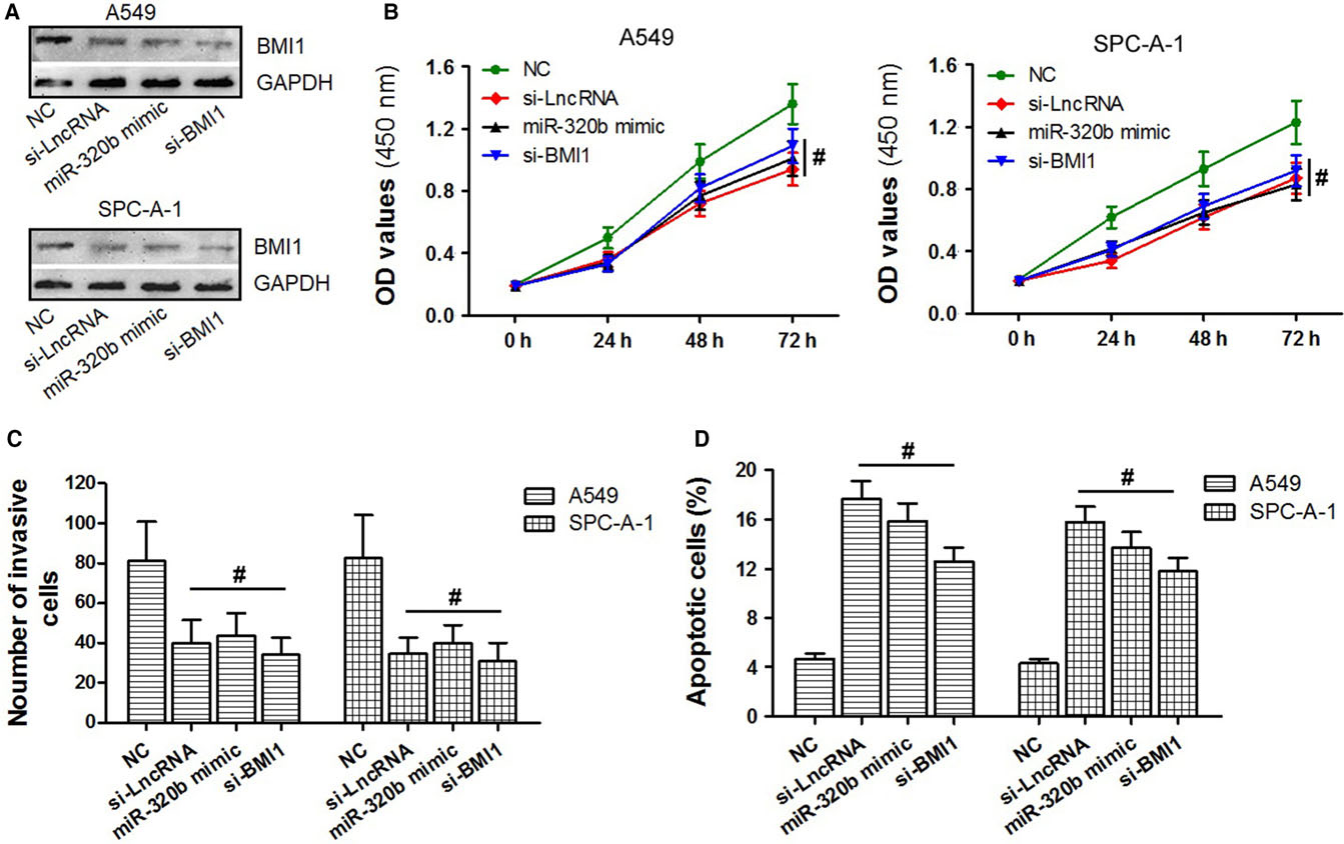
The effects of down‐regulated LncRNA NR2F2‐AS1, up‐regulated miR‐320b and si‐BMI1 of A549 and SPC‐A‐1 cells were compared. A, the BMI1 protein levels in si‐LncRNA NR2F2‐AS group, miR‐320b mimic group, si‐BMI1 group and NC group of A549 and SPC‐A‐1 cells within the use of Western blot assay. (B‐D) the condition of cell proliferation, cell invasion and cell apoptosis in si‐LncRNA NR2F2‐AS1 group, miR‐320b mimic group, si‐BMI1 group and NC group through the application of CCK‐8 assay, transwell detection and flow cytometry. ^#^ *P > *0.05

Through the study above, we learned that either down‐regulated LncRNA NR2F2‐AS or up‐regulated miR‐320b inhibited cell proliferation, invasion and induce cell apoptosis in A549 and SPC‐A‐1 cells. NR2F2‐AS could serve as the ceRNA through binding to miR‐320b. Besides, BMI1 is a direct target of miR‐320b. To further investigate the downstream mechanisms of LncRNA NR2F2‐AS in lung cancer cells, we also transfect A549 and SPC‐A‐1 cells with si‐BMI1 to compare the effects of si‐LncRNA NR2F2‐AS, miR‐320b mimic and si‐BMI1 among cell proliferation, invasion and apoptosis. As a result, the expression of BMI1 protein was completely suppressed in the three groups (si‐LncRNA NR2F2‐AS group, miR‐320b mimic group and si‐BMI1 group) when compared with the negative control group (*P < *0.05, Figure 6A). CCK‐8 assay, transwell assay and FCM assay were applied to detect the cell activity. As shown in Figure 6B, C, compared to the negative control group, the number of live cells and invasive cells in the three experimental groups was significantly less (*P < *0.05), on the contrary, the number of apoptotic cells in the three groups above was obviously increased (*P < *0.05, Figure 6D). Through the next statistical analysis among the three groups, there existed no difference in cells proliferation, invasion and apoptosis (*P > *0.05) suggesting that down‐regulated LncRNA NR2F2‐AS1, up‐regulated miR‐320b and si‐BMI1 shared similar effects on the activity of A549 and SPC‐A‐1 cells.

### 3.7 Down‐regulated miR‐320b or BMI1 lacking 3ʹUTR could reverse the effect of down‐regulated LncRNA NR2F2‐AS1 in A549 and SPC‐A‐1 cells

**Figure 7:**
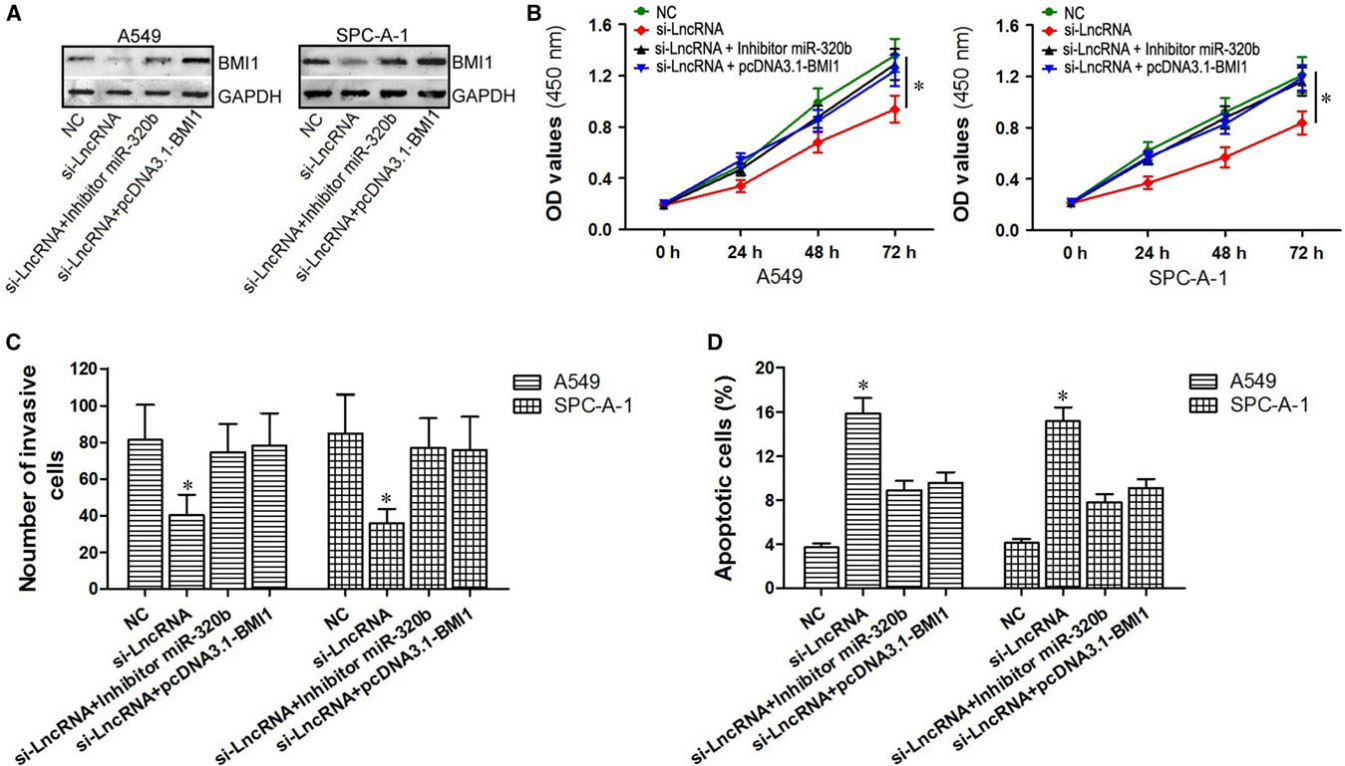
Down‐regulated miR‐320b or BMI1 lacking 3ʹUTR could reverse the effect of down‐regulated NR2F2‐AS1 in A549 and SPC‐A‐1 cells. A, representative Western blots showed BMI1 levels in si‐LncRNA group, si‐LncRNA+inhibitor miR‐320b group, si‐LncRNA+pcDNA3.1‐BMI1 group and NC group. (B‐D) the effects on cell proliferation, cell invasion and cell apoptosis in si‐LncRNA group, si‐LncRNA+inhibitor miR‐320b group, si‐LncRNA+pcDNA3.1‐BMI1 group and NC group through the application of CCK‐8 assay, transwell detection and flow cytometry. *P < 0.05

To determine whether NR2F2‐AS1 produce biological effects through modulating the regulation effects of miR‐320b on BMI1 in advance, rescue experiments were conducted. A549 and SPC‐A‐1 cells were divided into four groups respectively. The results of Western blot assay showed that in comparison with the NC group, the expression of BMI1 protein was obviously suppressed in si‐LncRNA group (*P < *0.05) but exerted no difference (*P > *0.05, Figure 7A) from the two cotransfection groups (si‐LncRNA+inhibitor miR‐320b group and si‐LncRNA+pcDNA3.1‐BMI1 group). On observing the results of the CCK‐8 assay (Figure 7B), we can find that only in the cells transfected with si‐LncRNA alone, cell proliferation was suppressed (*P < *0.05). On the contrast, when cells were cotransfected si‐LncRNA with miR‐320b inhibitor or pcDNA3.1‐BMI1, the condition of cell proliferation was similar to the NC group (*P > *0.05). When it comes to the effects on cell invasion and apoptosis, the number of invasive cells in si‐LncRNA group was significantly up‐regulated (*P < *0.05, Figure 7A) and the number of apoptotic cells was down‐regulated (*P < *0.05, Figure 7A) compared with those in the two cotransfection groups (si‐LncRNA+inhibitor miR‐320b group and si‐LncRNA+pcDNA3.1‐BMI1 group). These data suggested that down‐regulated miR‐320b or BMI1 lacking 3ʹUTR could reverse the effect of down‐regulated LncRNA NR2F2‐AS1 on the growth, metastasis and apoptosis of A549 and SPC‐A‐1 cells.

## 4 DISCUSSION

Long noncoding RNAs (lncRNAs) have access to participate in life activities within the organisms through various pathways. Han et.al proved that a long noncoding RNA protecting the heart from pathological hypertrophy.^26^ In multiple myeloma (MM) patients, LncRNA MEG3 played an essential role in osteogenic differentiation in bone marrow MSCs, partly by activating BMP4 transcription.^27^ Alterations in the function of lncRNAs promote tumour formation, progression and metastasis of prostate, bladder and kidney cancer; in this article, Martens‐Uzunova et  al also discussed the possible utilization of lncRNAs as novel biomarkers and potential therapeutic targets in urologic malignancies.^28^ In order to enrich the understanding of lncRNAs in NSCLC, our present study first confirmed the overexpression of LncRNA NR2F2‐AS1 among 39 cases of NSCLC tissues through qRT‐PCR. The expression of NR2F2‐AS1 was more up‐regulated in patients with advanced TNM stage or positive lymphatic metastasis, demonstrating that NR2F2‐AS1 may be involved in the progression of NSCLC. When it comes to cell test in vivo, the expression of NR2F2‐AS1 was also in higher level compared to that in NHBE cells. Secondly, the function of abnormal expression of NR2F2‐AS1 was discussed in A549 and SPC‐A‐1 cells. Si‐RNA against NR2F2‐AS1 was designed and transfected to the two cells to effectively down‐regulate the expression of NR2F2‐AS1. The results of flow cytometry, caspase3/7 detection, CCK‐8 assay, transwell assay and xenografts experiments in nude mice showed that down‐regulated LncRNA NR2F2‐AS1 contributed to the promotion of cell apoptosis and the inhibition of cell proliferation and invasion in A549 and SPC‐A‐1 cells in vivo and vitro. Then, we wonder how NR2F2‐AS1 functioned in NSCLC like this.

According to current research findings, the pool of mRNAs, lncRNAs and other non‐coding RNAs shared common MREs with miRNAs; lncRNAs thus could serve as natural miRNA “sponges” and inhibit miRNA function through competitive binding to MREs.^25^, ^29^ For example, knockdown of lncRNA NEAT1 inhibited glioma cell migration and invasion via the modulation of miR‐132.^30^ lncRNA‐H19 and miR‐141 could compete with each other and affect their target genes in gastric cancer.^31^ An lncRNA named CHRF was also found to act as an endogenous sponge of miR‐489, which down‐regulated miR‐489 expression levels as a result to regulate cardiac hypertrophy.^32^ In NSCLC patients, c‐Myc‐activated long non‐coding RNA H19 could down‐regulate miR‐107 and promote cell cycle progression.^33^ Then, in this study, we supposed there existed one or more miRNAs which could be regulated through the function of LncRNA NR2F2‐AS1. The bioinformatics analysis of microRNA database and the results of dual‐luciferase report assay showed that LncRNA NR2F2‐AS1 could act as a ceRNA by binding to miR‐320b. miR‐320b is significantly down‐regulated in several cancers including CRC,^34^ glioblastoma,^35^ gastric cancer^36^ and bladder cancer,^37^ playing a key role in plausible tumourigenesis. Our study first evaluated the role of miR‐320b in NSCLC. The expression of miR‐320b in 39 NSCLC patients was in a relatively low level, which is contrast to that of NR2F2‐AS1. When we artificially up‐regulate miR‐320b expression, the proliferation and invasion of A549 and SPC‐A‐1 cells were significantly suppressed, while the apoptotic cells were highly increased, demonstrating that up‐regulated miR‐320b inhibit the progression of NSCLC cells in cell proliferation, invasion and apoptosis to some degree.

As we all know, miRNAs could act as oncogenes or anti‐oncogenes within the modulation of various target genes, as a result, they could influence the transcription and translation of relative protein.^38^ For the sake of regulatory mechanisms of miR‐320b in NSCLC, the bioinformatics analysis of Target Scan and the results of dual‐luciferase report assay revealed that BMI1 is a direct target of miR‐320b. Besides, through Western blot assay, BMI1 protein expression was obviously inhibited when miR‐320b was up‐regulated, proving that miR‐320b could down‐regulate the expression of BMI1.

However, our results indicated that either down‐regulated NR2F2‐AS1 or up‐regulated miR‐320b inhibited cell proliferation, invasion and induced cell apoptosis in A549 and SPC‐A‐1 cells. NR2F2‐AS1 could serve as the ceRNA through binding to miR‐320b. Then, we supposed that miR‐320b may be affected by NR2F2‐AS1, and the signal pathway could be related to the effects of NR2F2‐AS1 in NSCLC. In order to prove this hypothesis, the effects on cell proliferation, invasion and apoptosis of A549 and SPC‐A‐1 cells among si‐LncRNA NR2F2‐AS, miR‐320b mimic and si‐BMI1 were compared through cellular experiments. The data revealed that cell activities in the three groups were similarly suppressed. The results of further rescue experiments also supported the assumption for they showed that the down‐regulated miR‐320b or BMI1 lacking 3ʹUTR could reverse the effect of down‐regulated NR2F2‐AS1 on the growth, metastasis and apoptosis of A549 and SPC‐A‐1 cells.

In conclusion, we described down‐regulated lncRNA NR2F2‐AS1 as an anti‐oncogenic lncRNA that contributed to the promotion of cell apoptosis and the inhibition of cell proliferation and invasion in A549 and SPC‐A‐1 cells through the regulation of miR‐320b/BMI1 axis. Based on the abnormal expression of NR2F2‐AS1 in NSCLC tissues, it may serve as a therapeutic target or useful marker in the treatment or diagnosis for NSCLC. For further understanding, more clinical and experimental studies need to be carried out.

## CONFLICT OF INTEREST

None.

## AUTHOR CONTRIBUTIONS

Shijie Zhang and Huaqi Wang designed and performed the cellular experiments, Shijie Zhang and Xiaoyun Zhang collected the tissues from patients, Xiaoyun Zhang and Qianqian Sun carried out parts of the experiments in vivo, Chunbo Zhuang, Guanlin Li and Li Sun also performed parts of cellular experiments and analysed the data, Qianqian Sun wrote the paper and participated in the manuscript revision.
